# Modelling the capacitance of the elongated plasma in tokamak

**DOI:** 10.1038/s41598-023-45503-7

**Published:** 2023-12-06

**Authors:** Pengcheng Miao, Ge Li, Song Zhang, Zhiyuan Weng, Yu Wu, Zemin Duan

**Affiliations:** 1 Institute of Energy, Hefei Comprehensive National Science Center, Hefei, 230031 Anhui People’s Republic of China; 2 Institute of Plasma Physics, Chinese Academy of Sciences (ASIPP), Hefei, 230031 Anhui People’s Republic of China; 3 School of Electronic Information Engineering, China West Normal University, Nanchong, 637002 Sichuan People’s Republic of China; 4 School of Nuclear Science and Technology, University of Science and Technology of China, Hefei, 230029 Anhui People’s Republic of China; 5 School of Electrical and Optoelectronic Engineering, West Anhui University, Lu’an, 237012 Anhui People’s Republic of China; 6 Hefei Hangtai Electro Physics Co., Ltd, Hefei, 230031 Anhui People’s Republic of China

**Keywords:** Nuclear fusion and fission, Energy science and technology

## Abstract

The capacitance model suitable for the non-circular cross-section plasma is studied based on the capacitance model of the circular cross-section plasma. The coaxial elliptic-torus capacitor property is further derived and used to determine the capacity of non-circular cross-section tokamak plasma, such as EAST (Experimental Advanced Superconducting Tokamak). By testing all the physical terms in this model, we find that the capacitance $Cp$) is increasing exponentially with the increase of elongation ratio (*k*_2_/*k*_1_), while the minor radius ratio (*a*_2_/*a*_1_) is just reversed at the flat-top of plasma current, and the capacitance property is implicitly included in the H-mode study during the L–H transition. It is noted that *C*_*p-H mode*_ is the least and *C*_*p-I mode*_ is approximately equal to *C*_*p-L mode*_ under the L-mode, I-mode and H-mode regimes based on this capacitance model in EAST. Consequently, it may be integrated into an equivalent circuit of the tokamak transformer or transport computer code of the edge plasma for use in precise simulations of fusion plasma behavior in the future, such as ITER (International Tokamak Experimental Reactor) or BEST (Burning-plasma Experimental Superconducting Tokamak) in China.

## Introduction

Fusion energy is a promising source of clean energy. Physicists have developed a keen interest in nuclear fusion as they study the formation and evolution of the sun. Nuclear fusion reactor power plants will enable humans to take advantage of the stellar energy while eliminating or substantially reducing the disadvantages of other energy sources, such as energy shortages and environmental pollution^1,2^. In a reactor, the product of ion density, energy confinement time, and temperature must be in the right parameter range. The required value of the product is approximately 5 × 10^21^ m^−3^ s keV. Now, although the required temperature, density, and confinement time have all been obtained in tokamaks, the design of such a reactor raises a wide range of questions. A commercial reactor even more so. Therefore, fusion energy still has a long way to study before it can be commercialized^3,4^. Tokamak is a toroidal magnetic plasma confinement device, which is an extreme-complex electromagnetic system and the leading candidate among the most promising paths for producing fusion energy. In essence, it is the pulsed devices modelled as a toroidal transformer with one turn secondary plasma ring circuit coupled with a primary poloidal field and a central solenoid coils circuit based on the principles of electrotechnology^5–7^. To achieve really steady-state confinement, superconducting magnets are the most effective way to validate the engineering feasibility of a tokamak for fusion energy application. EAST is the first fully superconducting tokamak, which has the ITER-like magnetic configurations, to demonstrate high performance and steady state operation^8,9^. The experiments can contribute to improve the scientific basis for ITER and such a fully superconducting tokamak is urgently needed for the development of a future tokamak fusion reactor^10^. The ITER is configured as a next-generation tokamak machine that is now the flagship facility for the magnetic confinement fusion (MCF) society and its safety and electrical parameters are concerned by all ITER party members across the world^11,12^. The value of capacitance for ITER will be dramatically high because of its large size and high-density operation regime and could be a key parameter and should be considered. We assume that a dielectric medium (diluted plasma) exists between the plasma and the tokamak chamber, where it leads to a capacitance. The value of capacitance is one of the key factors for the transformer model of tokamaks. It may play an important role in tokamak physics research. However, it has not yet been seriously investigated and considered in tokamak plasma except in a preliminary study of disruption instability and fundamental properties based on a coaxial cable capacitance in the Damavand tokamak with the circular cross-section plasma^13^. It is apparent that the estimation of the value of the capacitance based on a coaxial cable capacitance model does not apply to the cases of non-circular cross-section plasma, such as EAST, ITER and BEST. Consequently, we have developed the coaxial elliptic-torus capacitance model to calculate the capacitance for the elongated plasmas.

## Results

**Figure 1:**
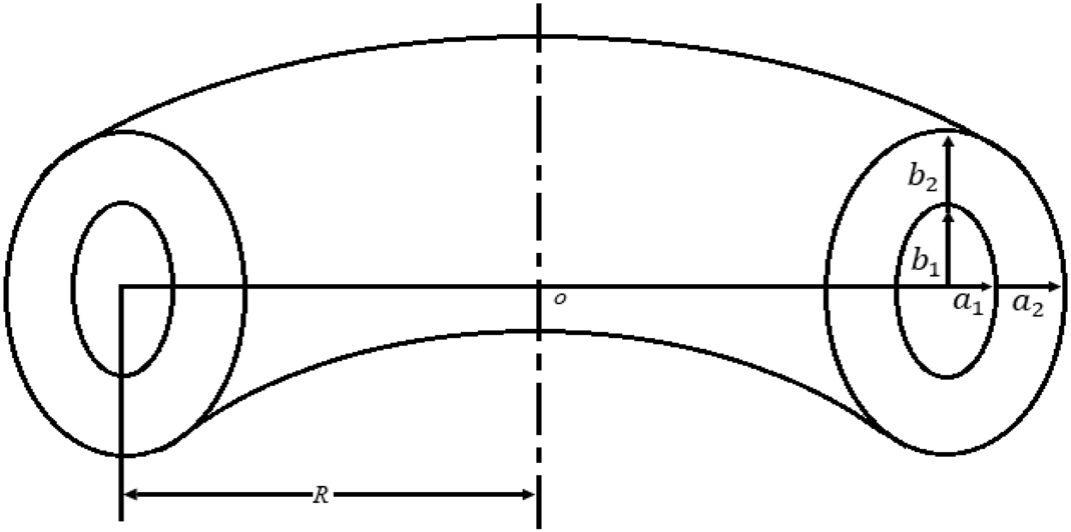
Coordinates in the coaxial and concentric elliptic-torus geometry.

In this study, a complete model is constructed for a non-circular cross-section elongated plasma to calculate the capacitance with a clear logical mechanism: the elliptic cross-section of an elliptic torus capacitor is transformed to a circular cross-section by simple conversion. Then, the capacitance model of the coaxial cable ring is deduced by using the formula of the circular cylindrical capacitor to realize the calculation of the capacitance of the elliptical torus in Fig. 1. ${a}_{1}$(${a}_{2}$) and ${b}_{1}$(${b}_{2}$) are the semi-major axis length and semi-minor axis length of ellipses, respectively. We get the capacitance of the elongated plasma configuration as

1 $${C}_{p}=4{\pi }^{2}\varepsilon R\left[\frac{1+{\left(\frac{c}{R}\right)}^{2}\times \left(\frac{{k}_{2}^{2}-{k}_{1}^{2}}{{(k}_{1}^{2}-1)({k}_{2}^{2}-1)}\right)}{0.5\times \mathrm{ln}\left({(k}_{1}^{2}-1)/{(k}_{2}^{2}-1\right))}+\frac{2{c}^{2}}{{R}^{2}\sqrt{{k}_{1}^{2}-1}\times \sqrt{{k}_{2}^{2}-1}}\right],$$

where *R* is the major radius of the torus, $c$ is the semi-focus length of ellipses, ${k}_{1}$ and ${k}_{2}$ are respectively the elongations of inner and outer ellipse.

The data from the typical discharge of the EAST tokamak can be used to study the correctness of this model and analyze the relationship of between the plasma capacitance and the radial electric field of the edge plasma under the different cases, such as L/L–I/L–H mode transitions. Combined with earlier studies of plasma inductance *L*_*p*_^12^, it is interesting to note that the computed coherent frequency of plasma inductance *L*_*p*_ and its capacitance is the central frequency of weakly coherence mode (WCM) as measured in Refs.^14,15^ on EAST. Calculation results clearly showed that this capacitance model is accurate and reliable. The plasma capacitance of EAST is significant and of order ∼10^−6^ F and comparable with the approximated values of the capacitances on TEXTOR, JT60U, and JET in Ref.^13^. Moreover, the capacitance value of H-mode becomes less than that of L-mode and I-mode. The plasma capacitance has experienced a sharp decrease during the period of L–H mode transition, in accordance with the radial electric field ${E}_{r}$ change as measured by Reciprocating Langmuir Probe i.e. RLP at the outer midplane in SOL. Therefore, the capacitance property is implicitly included in the H-mode study. These findings indicate that the value of this capacitance is significant for tokamaks and may play an important role in tokamak research, especially for the ITER.

## Discussion

It can be assumed that diluted plasma is regarded as a dielectric medium between the plasma and the first wall of the chamber. The capacity of non-circular cross section plasma can be done by taking into consideration a capacitor made up of a concentric elliptic torus on EAST, as shown in Fig. 1. The elongated plasma capacitance is calculated based on the model of a coaxial-torus capacitance property and coaxial cables with Roucofeskie’s conversion. As the key characteristics of a coaxial cable are its inductance, resistance, capacitance, and effective shunt conductance, the tokamak plasma would also have these characteristics of a coaxial torus, as verified by the discharge parameters of the Damavand tokamak, so it is reasonable to calculate the capacitance of an elongated plasma configuration by using the above method.

**Figure 2:**
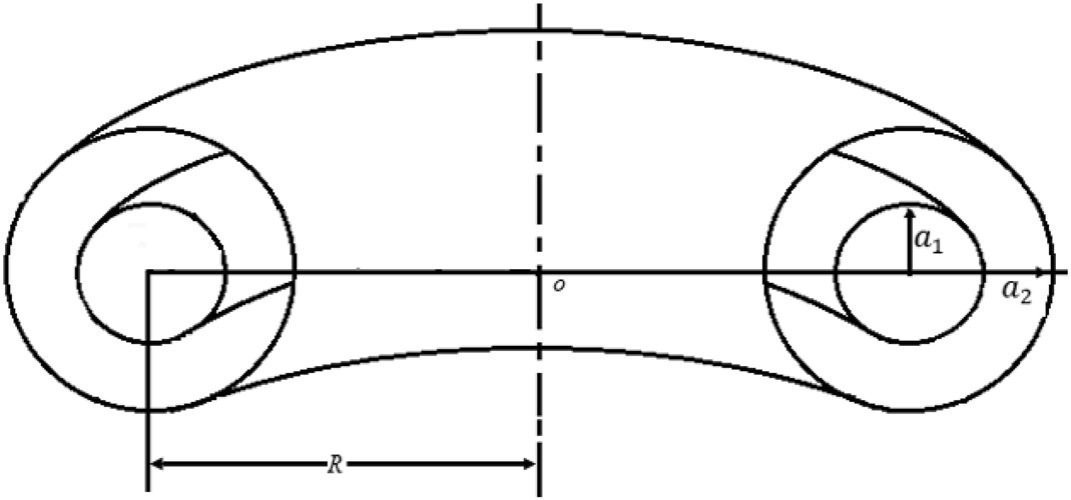
Coordinates in the coaxial-torus geometry.

The coordinate system of a toroidal geometry coaxial capacitor is selected to take advantage of azimuthal symmetry and to facilitate the application of boundary conditions, as shown in Fig. 2. The radius of the outer torus (minor radius of the tokamak vacuum chamber) is* a*_*2*_ while the radius of the cross section of the inner torus (plasma minor radius) is *a*_1_. The torus’s (tokamak’s) main radius is* R*. The capacitance of the circular plasma is derived as^13^,

2 $${C}_{p}=4{\pi }^{2}\varepsilon R\left[\frac{1+{\left(\frac{{a}_{1}}{R}\right)}^{2}-{\left(\frac{{a}_{2}}{R}\right)}^{2}}{\mathrm{ln}\left(\frac{{a}_{2}}{{a}_{1}}\right)}+\frac{2{a}_{1}{a}_{2}}{{R}^{2}}\right].$$

In the confocal elliptic torus,

3 $$c={c}_{1}=\sqrt{{b}_{1}^{2}-{a}_{1}^{2}}={c}_{2}=\sqrt{{b}_{2}^{2}-{a}_{2}^{2}} ,$$

and assuming ${k}_{1}={b}_{1}/{a}_{1},{k}_{2}={b}_{2}/{a}_{2}$ (i.e. the elongations of tokamak plasma and vacuum vessel respectively), we get

4 $${a}_{1}=\frac{c}{\sqrt{{k}_{1}^{2}-1}}, {a}_{2}=\frac{c}{\sqrt{{k}_{2}^{2}-1}}.$$

Combining (2), (3) and (4), the capacitance of non-circular cross-section plasma is generalized as Eq. (1).

The permittivity of the dielectric *ε* is one of the critical parameters for the calculation of tokamak plasma capacitance. Since the current and voltage in the capacitance is related to the radial component, we will use the perpendicular dielectric constant (${\varepsilon }_{\perp }$) by^16^

5 $$\varepsilon ={\varepsilon }_{\perp }{\varepsilon }_{0}, {\varepsilon }_{\perp }=1+\left({c}_{d}^{2}/{v}_{A}^{2}\right) , {v}_{A}={B}_{T}/{({\mu }_{0}\rho )}^{1/2} ,$$

where ${\mu }_{0}$ is the permeability of free space, $\rho$ the specific mass and ${c}_{d}$ the velocity of light. It might be a valid estimate to just take into account the lowest value of ${\varepsilon }_{\perp }$ since in series capacitances (1/*C*_*p*_ = 1/*C*_1_ + 1/*C*_2_ + $\cdots$) the lowest one is dominant, so it is a reasonable assumption to take the ${\varepsilon }_{\perp }$ which is determined by the condition of the region between the scrape-off layer and the chamber wall as a dielectric, similar to that of circular plasmas^13^. This medium is usually a diluted and low-density plasma, which decreases exponentially.

It is well known that the tokamak plasma has ohmic and induction properties. For elongated plasma from above the non-circular cross-section, the total plasma inductance is thus derived as^12,17^

6 $${L}_{p}={\mu }_{0}R[\mathrm{ln}\left(8R/a\sqrt{k})+{l}_{i}/2-2\right].$$

In the EAST tokamak, stationary I-mode is also identified by the weakly coherent mode (WCM)^14,15^. The WCM corresponds to electron turbulence, leading to L-mode–like particle transport. The coherent frequency may be calculated by the plasma inductance and plasma capacitance.

## Methods

### Verification of the elongated plasma capacitance model in EAST

EAST is a superconducting tokamak with major radius *R* = 1.85 m, minor radius* a* = 0.45 m, maximum plasma current *I*_*p*_ ≤ 1MA, toroidal magnetic field *B*_*T*_ ≤ 3.5* T*, elongation *k* = 1.2–2 and plasma pulse length already tested up to over 1000 s with high-confinement super I-mode at H_98y2_ ~ 1.2^15^, the I-modes are free of disruptive Edge Local Modes, i.e. ELM.

**Figure 3:**
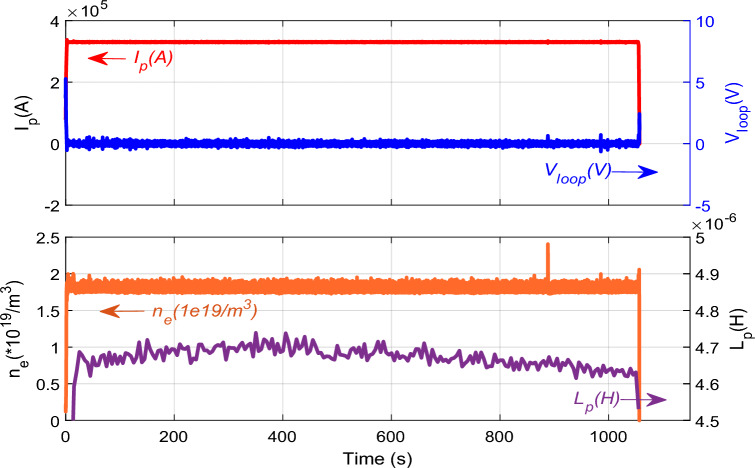
Evolution of plasma parameters of the super I-mode discharge EAST #106915. From top to bottom: plasma current (*I*_*p*_), loop voltage (*V*_*loop*_), line-averaged electron density (*n*_*e*_), and plasma inductance (*L*_*p*_).

In order to check the accuracy and validity of the developed capacitance formula, Fig. 3 shows the results of the plasma capacitance value and some experimental parameters of super I-mode plasma discharge #106915 in EAST. The plasma was operated at *I*_*p*_ = 330 kA, *n*_*e*_ = 1.8 × 10^19^ m^−3^ and *B*_*t*_ = 2.75 T; it was heated by a total RF power of 1.65 MW (1.1 MW of LHCD at 4.6 GHz and 0.55 MW of ECRH)^15^. The WCM has been regarded as an indicator of the appearance of the I-mode, and obtained from the power frequency spectrum of the time derivative of the density fluctuation phase, measured using a Doppler reflectometer (DR) at the normalized radius *ρ* = 0.91. In this study, the frequency is also calculated by the plasma capacitance (1) and the plasma inductance (6). They are *L*_p_ ~ 4.68 × 10^−6^ H and *C*_p_ ~ 1.91 × 10^−6^ F at 90 s, respectively. The frequency is about *f* ~ 53 kHz by $f=1/(2\pi \sqrt{{L}_{p}\times {C}_{p}})$. It sits just between 30 to 100 kHz, the typical frequency regime of dominant turbulence mixed with that of WCM^14,15^.

### Analysis to the capacitance on EAST

**Figure 4:**
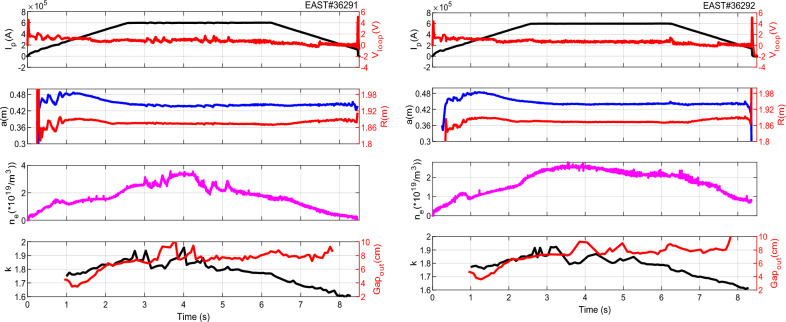
Result of EAST #36291 and #36292 shot-the typical LHCD wave alone L/H-Mode discharge. From top to bottom: plasma current and loop voltage, major and minor radius, line averaged density, plasma elongation and the outboard gap.

In addition, we calculated the plasma capacitance values of (1), (2) and (5) under the different cases (L/L–H mode transition) based on the typical discharge shots of EAST #36291 and #36292. In the typical L/L–H mode transition discharge experiment shots of EAST #36291 and #36292, with lower hybrid current drive (LHCD) wave only, the critical parameter evolutions are presented in Fig. 4.

**Figure 5:**
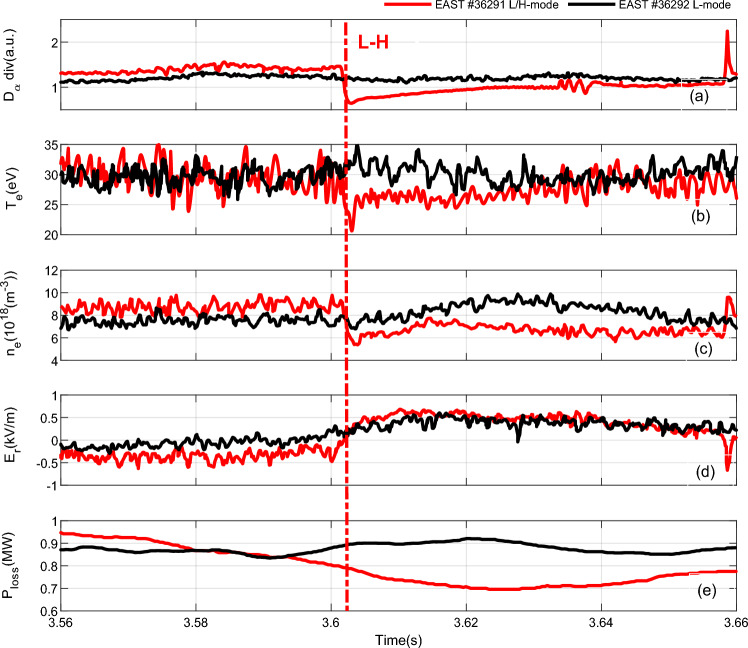
Two adjacent shots of EAST #36291 and #36292 at 3.56–3.66 s by RLP measurements at the outer midplane in SOL. From top to bottom:${D}_{\alpha }$, ${T}_{e}$, ${n}_{e} ,$ the radial electric field ${E}_{r},$ and ${P}_{loss}$ in Ref.^18^.

**Figure 6:**
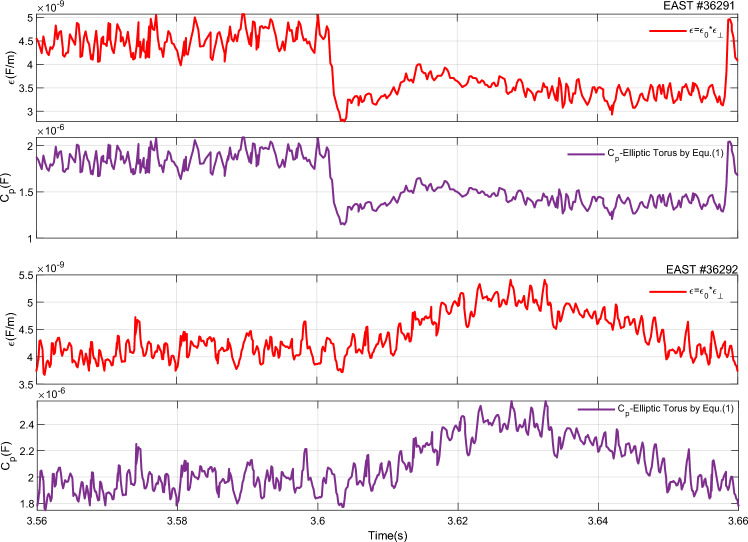
Time traces of the perpendicular permittivity and the plasma capacitance in EAST #36291 and 36292.

They were all maintained for ~ 8.5 s, and almost contained the same experimental parameters. At the flat-top phase, the plasma current of the two discharges was ~ 600 kA and the loop voltage were well-controlled at a value that was almost equal to 0 V, meaning that almost all the plasma current was driven noninductively. In this work, the fast-RLP probe system was used to provide a direct measurement of the parameters from the wall to the plasma in the scrape-off layer region (SOL). The probes were put at the same position in two adjacent shots. Figure 5 shows the measured results of the two adjacent shots at 3.56–3.66 s, the red line stands for the H-mode discharge with shot number 36291, and the black line for L-mode discharge with shot number 36292. *P*_*loss*_ is defined by Eq. (7) according to

7 $${P}_{loss}={P}_{OH}+{P}_{LH}-{P}_{rad}-dW/dt,$$

where ${P}_{OH}$ is the Ohmic power; ${P}_{LH}$ is the net LHW power; ${P}_{rad}$ is radiation power; *W* is the stored energy. For the Eq. (1), the perpendicular permittivity ${\varepsilon }_{\perp }$ is the critical parameter, which determined by the condition of the region between the scrape-off layer and the chamber wall as a dielectric. Because of the different density profile, ${\varepsilon }_{\perp }$ is highest in the center of the plasma and the area between the plasma edge and the chamber wall is the lowest. The perpendicular permittivity can be calculated by using the experimental result of electron density in scrape-off layer by RLP. Thus, we obtain ${C}_{p}$ by Eq. (1) as shown in Fig. 6. Obviously, it is significant and of order ∼ 10^−6^ and comparable with the approximated values of the capacitance for TEXTOR, JT60U, Damavand, and JET with about *C*_*p*_ ∼ 1.9 × 10^−6^ F, 2.5 × 10^−6^ F, 1.7 × 10^−6^ F and 6.8 × 10^−6^ F respectively^13^.

Figure 6 illustrates the time histories of the ${C}_{p}$ and its associated plasma perpendicular permittivity. It can be seen that the plasma capacitance value of H-mode is much less than that of L-mode discharge in Fig. 6, which also indicates that the plasma confinement is enhanced. For EAST #36291 shot, the plasma capacitance has experienced a sharp decrease during the period of L–H transition with about ${C}_{p{\text{-}}L\, mode}$∼1.8 × 10^–6^ F and ${C}_{p{\text{-}}H \,mode}$∼1.4 × 10^–6^ F while for EAST #36292 shot with about ${C}_{p{\text{-}}L\, mode}$∼1.9 × 10^–6^ F. In addition, it is nearly equivalent to each other at L-mode for #36291 and #36292 shots. It can be explained as follows. In H-mode #36291 shot, the plasma capacitance decreases with the decreasing electron density in the SOL compared with that of the L-mode shot #36292. This leads to a reduction in the level of particle recycling at the plasma edge region. Accordingly, the energy loss (*P*_*loss*_) decreases and the storage energy increase gradually, so the plasma energy confinement is improved, as shown in Fig. 5e. The plasma capacitance has a peaking point at 3.63 s for the EAST #36292 shot in Fig. 6—the reason is that the perpendicular permittivity, which has increased remarkably with the plasma density.

It has been proved by many experiments that the emergence of the negative electrical field at the edge of plasma is an important character of L–H mode transition. It is a key parameter for improving plasma confinement in H-mode physics. ${D}_{\alpha }$, ${T}_{e}$, ${n}_{e}$ in the SOL decreased rapidly over the time scale of about 1 ms, and mean-while, the radial electric field ${E}_{r}$ turned positive in Fig. 5. The net charge appears owing to ion loss at the edge of tokamak plasma. Spatial distribution of the net charge produces a radial electric field. The electric field will vary with the plasma elongation *k*_2_ and the electron density (i.e. the net charge), yet the plasma capacitance *C*_*p*_ is a physical quantity related to them in Eqs. (1) and (5). Therefore, the radial electric field could be related to the plasma capacitance here under L–H mode transition discharge.

### Effect of* k* on the capacitance

**Figure 7:**
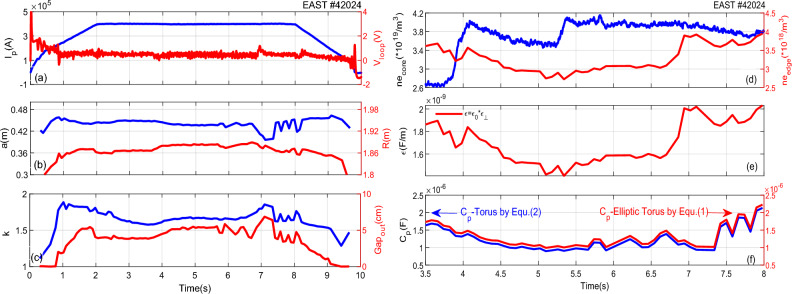
Time traces of the typical waveforms of the H-mode discharge in EAST#42024.

The elongation *k* of divertor plasma (the elongation range being 1.6–2.0 in EAST) is the key parameter to determine the capacity of non-circular cross-section plasma, which is different from circular cross-section plasma. The effect of *k* on the capacitance is investigated further with plasma current *I*_*p*_ ~ 0.4MA, major radius *R* ~ 1.88 m, minor radius* a* ~ 0.45 m, elongation *k* ~ 1.6–1.8 for H-mode discharge in EAST #42024. The typical waveforms are shown in Fig. 7.

As seen from the calculated capacitance values by (1) and (2) during H-mode, the ${C}_{p}$ is higher based on (1). What is more, one can see a good coincidence in the changing trend for ${C}_{p}$ and *ε, n*_*e-edge*_ during 3.5–6 s since the minor radius *a, R, k* and *Gap*_out_ remain nearly constant in Fig. 7d–f. However, ${C}_{p}$ is changed in accordance with $a$, while the *Gap*_out_ is just the reverse from 6 to 8 s in Fig. 7b,c,f. Therefore, it can be concluded that ${C}_{p}$ depends on the minor radius and *Gap*_out_ of plasma at the flat-top of current from the experimental results in Fig. 7 and the calculation by Eqs. (1) and (5). The minor radius (*a*_1_, *a*_2_) and elongation (*k*_1_, *k*_2_) are the main variables of Eq. (1) in the capacitance model. The qualitative rules of the capacitance changing with *a* and *k* are also analyzed for the sake of simplicity.

**Figure 8:**
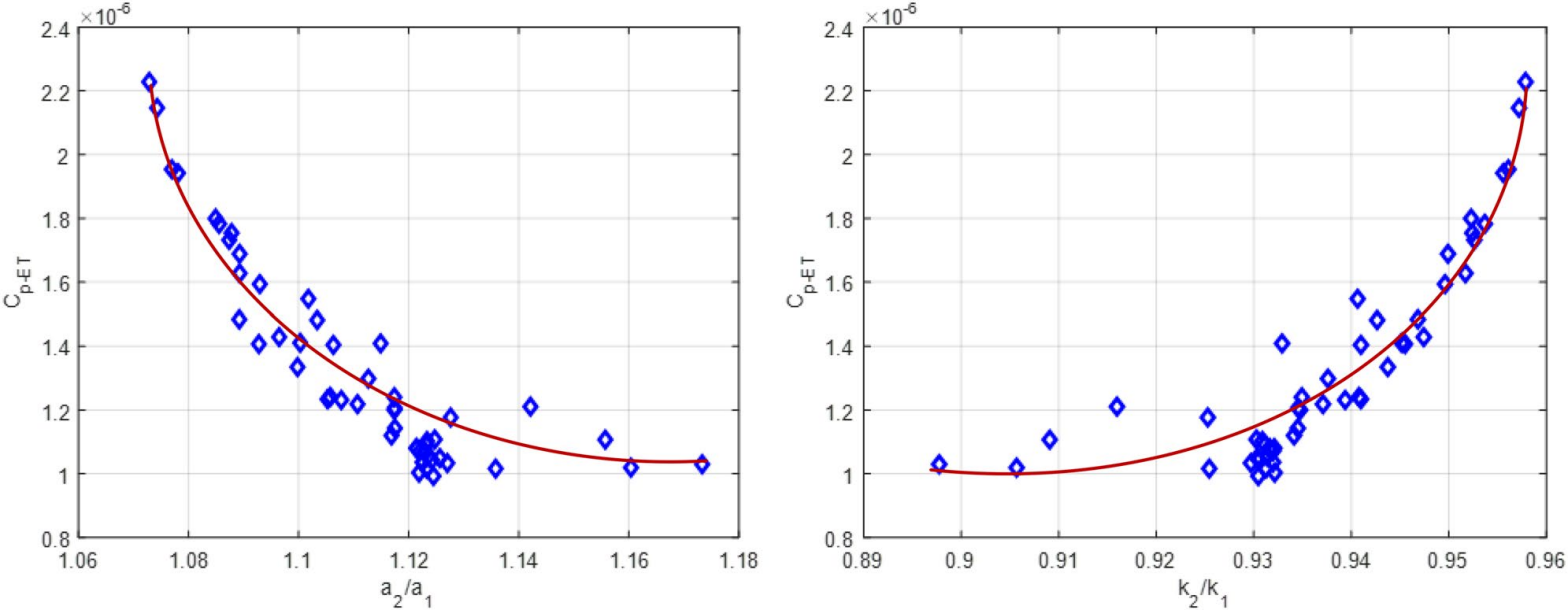
The plasma capacitance ${C}_{p{\text{-}}ET}$ by varying minor radius *a*_*2*_/*a*_*1*_ and elongation* k*_*2*_/*k*_*1*_ in EAST #42024.

**Figure 9:**
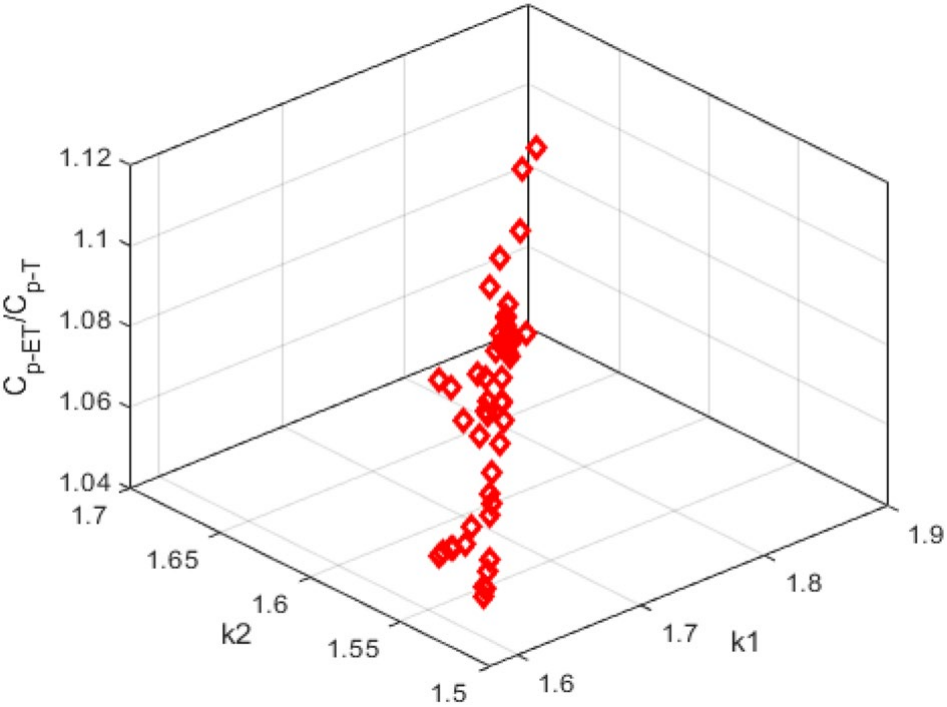
The ratio ${C}_{p{\text{-}}ET} /{C}_{p{\text{-}}T}$ of plasma capacitance by varying elongation *k*_1_ and *k*_2_ in EAST #42024.

As shown in Fig. 8, it turns out that the capacitance (${C}_{p{\text{-}}ET}$) increases exponentially with increasing elongation ratio (*k*_*2*_/*k*_*1*_) in Fig. 8b, while for the* a*_*2*_/*a*_*1*_*,* the capacitance (${C}_{p{\text{-}}ET}$) is just reversed in Fig. 8a. In addition, the ${C}_{p{\text{-}}ET} /{C}_{p{\text{-}}T}$(i.e. the deviation of capacitance value) is also plotted to investigate further the effect of elongation on plasma capacitance ${C}_{p{\text{-}}T}$(circular torus) by (2) and ${C}_{p{\text{-}}ET}$(elliptic torus) (1) in Fig. 9. Obviously,${C}_{p{\text{-}}ET} /{C}_{p{\text{-}}T}$ seem to increase significantly with increasing *k*_1_ and *k*_2_ in EAST #42024. This indicates that Eq. (1) considering the plasma elongation *k* is an available method and obviously better than (2) to calculate the ${C}_{p}$ value of non-circular cross-section plasma discharges in EAST.

### Comparison of the L/I-mode plasma capacitance

**Figure 10:**
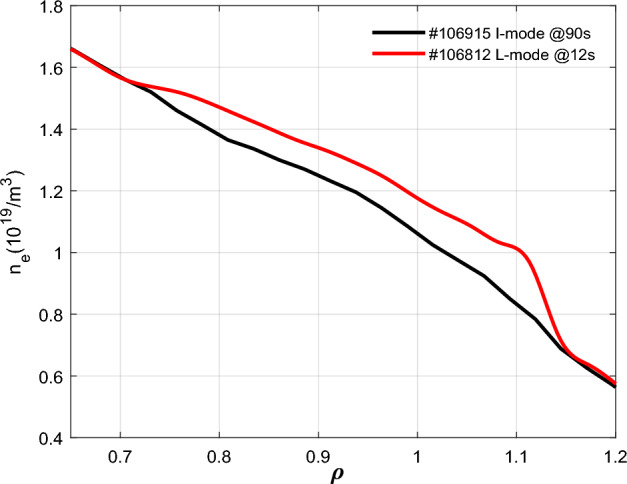
The edge electron density profiles from reflectometry in EAST #106915 and #106812.

The I-mode is a stationary high energy confinement regime that has been a focus of increasing interest and exploration in recent 10 years. It combines the advantages of both the H-mode and L-mode regimes. For the thousand seconds of I-mode plasma discharge achieved on EAST (#106915), the H_98_ factor is higher than 1, the same level of energy confinement as H-mode^15^. It is similar to the L-mode discharge (#106812) in the heating power, plasma current and line-averaged density. Here, the capacitance is further calculated and compared to the L-mode (#106812) plasma. Figure 10 displays the electron density profile measured by Reflectometry of two shots in I-mode and L-mode, respectively^15^.

The capacitance (*C*_*p*_) and associated plasma parameters based on (1) are listed in Table 1 for the EAST #106915 and #106812 shots. Note that the qualitative relationship between variables, such as *a*, *k*, *Gap*_*out*_, and *C*_*p*_, is in agreement with that of the EAST #42024 shot as mentioned in section (*c*). Moreover, unlike the *C*_*p-H mode*_, the *C*_*p-I mode*_ and *C*_*p-L mode*_ are nearly equivalent to each other in Table 1. It may be due to the fact that the particle confinement of the I-mode plasma remains almost identical to that in the L-mode. So the electron density and the perpendicular permittivity of I-mode plasma are similar to those of L-mode discharge (#106812) in the SOL, as shown in Fig. 10 and Table 1. Therefore, it may be concluded from the results that *C*_*p-H mode*_ is the least and *C*_*p-I mode*_ is approximately equal to *C*_*p-L mode*_ under L-mode, I-mode and H-mode plasma discharges in EAST.

**Table 1:** Measured and calculated plasma parameters of EAST #106915@90 s and #106812@12 s.

EAST-Shot	*R* (*m*)	*a* (*m*)	*k*	*Gap*_*out*_ (*m*)	$\varepsilon$(*F/m*)	*C*_*p*_(*F*)
#106812(L-mode)	1.8985	0.4530	1.7569	0.0455	2.7670 × 10^–9^	2.1529 × 10^–6^
#106915(I-mode)	1.9059	0.4566	1.7264	0.0411	2.1430 × 10^–9^	1.9110 × 10^–6^

## Conclusions

In summary, the long-pulse steady-state operation at the H-mode is the crucial goal of current fusion energy research. Here, a plasma capacitance model suitable for the non-circular cross-section plasma is developed and validated based on the typical plasma discharges over all confinement regimes (L-mode, I-mode, H-mode) in EAST. The model could lead to the following results: (1) The capacitance of the typical plasma discharge is significant, with about of ∼10^−6^ F and increasing exponentially with the increase of elongation ratio (*k*_2_/*k*_1_), while for the* a*_2_/*a*_1_, it is just reversed. (2) The capacitance of H-mode discharge is the least and *C*_*p-I mode*_ is approximately equal to *C*_*p-L mode*_ under the L-mode, I-mode and H-mode confinement regimes based on this capacitance model, which also indicates that the plasma confinement is enhanced in H-mode discharge, compared with the L-mode and I-mode. (3) The capacitance has experienced a sharp decrease during the period of L–H transition, in accordance with the radial electric field ${E}_{r}$ change as measured by RLP at the outer midplane in SOL. Therefore, the capacitance property is implicitly included in the studies of H-mode and I-mode. Particularly the super I-mode with WCM is already tested experimentally to be sustained over 1000 s in EAST at H_98y2_ ~ 1.2^15^, the ELM-free mode paves an experimentally gentle path for ITER and BEST.

## Data Availability

The datasets analyzed during the current study available from the corresponding author on reasonable request.
